# Effects of primary calcium source on phytase efficiency in weaned piglets

**DOI:** 10.1093/jas/skaf396

**Published:** 2025-11-12

**Authors:** Xiaonan Guan, Adam Smith, Hengxiao Zhai, Francesc Molist, Liz Vanessa Lagos

**Affiliations:** Schothorst Feed Research, 8200 AM Lelystad, The Netherlands; dsm-firmenich Nutritional Products (UK) Ltd, Heanor, Derbyshire, United Kingdom; dsm-firmenich Animal Nutrition Research Center, Bazhou, China; Schothorst Feed Research, 8200 AM Lelystad, The Netherlands; Schothorst Feed Research, 8200 AM Lelystad, The Netherlands

**Keywords:** bone ash, calcium formate, limestone, mineral digestibility, phytase, weaned piglets

## Abstract

An 18-d experiment tested the hypothesis that using calcium formate (Ca-formate) instead of limestone in piglet diets, lowers the dietary buffering capacity, reduces stomach pH, and improves phytase efficiency. At weaning (29.8 ± 1.10 d), 60 boars (body weight 8.8 ± 0.31 kg) housed in metabolism crates were allotted to a randomized complete block design with 10 experimental diets and 2 weaning rounds. Diets were formulated following a 5 × 2 factorial design with 5 dietary treatments (Trt) and 2 main Ca sources (limestone or Ca-formate). The 5 Trt included a positive control diet [PC; 0.76% Ca, 0.34% standardized total tract digestible (STTD) P] and 4 negative control diets (NC; 0.56% Ca, 0.21% STTD P) supplemented with phytase at 0, 750, 1,500, and 3,000 FYT/kg. At day 10, piglets were individually housed to allow fecal and urine sample collection. On days 0, 7, and 18, blood samples were collected. At day 18, samples of gastrointestinal content and the metacarpus were collected, and apparent jejunal, ileal, and total tract digestibility (AJD, AID, and ATTD) of Ca and P was calculated. Digestibility and blood data were analyzed as a 2-way and 3-way interaction, respectively. Phytase dose-response regressions for ATTD Ca and P concentrations were performed per Ca source. Regardless of Trt, Ca source did not affect stomach pH, but Ca-formate diets had greater AID and ATTD of Ca (+5.7% and 3.6%; *P* < 0.01) and lower AID and ATTD of P (−3.2% and 2.4%; *P* < 0.01) than limestone diets. Increasing phytase levels increased the ATTD and AID of Ca and P (*P* < 0.01). An interaction between Trt and Ca source was observed for AJD of P (*P* < 0.01). There was no effect of Ca source, except at 750 FYT, where the Ca-formate diet had lower AJD of P than the limestone diet. Based on the dose-response analyses, to reach a dietary ATTD P of 3.84 g/kg, 1,232 and 1,944 FYT are needed in the limestone and Ca-formate diets, respectively. Compared with the NC, increasing levels of phytase and the PC diet increased serum P (mg/L) and P retention (%), phytase inclusion increased plasma inositol (µmol), but only the PC diet increased bone ash (g/kg; Trt main effect; *P *< 0.001). In conclusion, replacing limestone with Ca-formate does not lower stomach pH, increases Ca but reduces P digestibility, and decreases phytase efficiency, likely due to greater Ca solubility, which induces the formation of insoluble Ca-P-phytate complexes. This highlights the importance of considering a digestible Ca system in piglet diets.

## Teaser

Using calcium formate to replace limestone in piglet diets increases the digestibility of Ca, but decreases the digestibility of P. The efficiency of phytase is also compromised by calcium formate, highlighting the importance of considering the digestibility of Ca when formulating piglet diets.

## Graphical Abstract

**Graphical Abstract:**
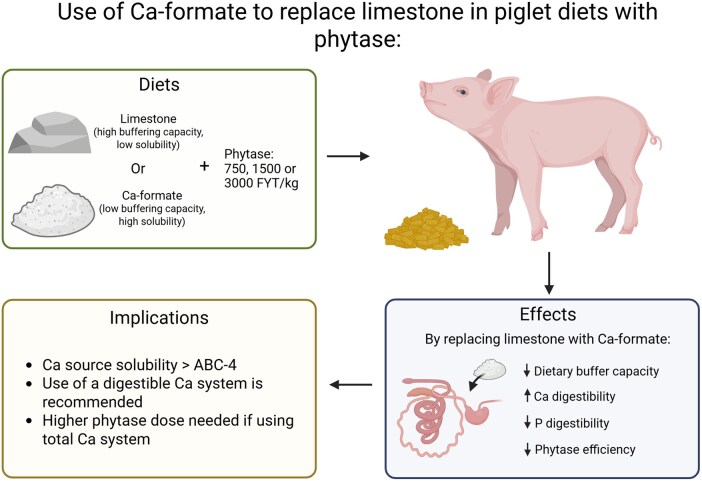

## Introduction

In the last decade, Ca has received much attention for pig diet formulation due to the influence that the dietary Ca to P ratio has on growth performance and the clear negative effect that excess Ca exerts on P utilization and animal performance (Letourneau-Montminy et al., 2012). This has motivated a number of experiments dedicated to determining the digestibility of Ca in different feed ingredients and establishing digestible Ca requirements for pigs, with the ultimate goal of moving toward a digestible Ca system that helps formulate pig diets more accurately (Lee et al., 2023). Most of the digestibility studies have focused on limestone as it is the most commonly used Ca supplement in the swine industry. However, the high acid binding capacity (ABC) of limestone, along with the underdeveloped capacity of gastric acid secretion in weaned piglets, have led to the search for alternative Ca sources with lower buffer capacity in weaner diets (Huting et al., 2021). Calcium formate (Ca-formate) has become a product of interest to partially or fully replace limestone in piglet diets, especially in Europe.

The ABC is the ability of a diet or ingredient to resist a change in pH and it is calculated as the amount of acid or base (mEq) needed to reach a target pH value, usually 4 (ABC-4; Huting et al., 2021). This value is not only relatively easy to obtain but it is also considered additive in complete diets (Lawlor et al., 2005; Batonon-Alavo et al., 2016), allowing diet formulation to target ABC-4 values through careful selection of feed ingredients. The ABC-4 of Ca-formate is around 3,983 mEq/kg, whereas for limestone, the average ABC-4 is three times greater, 12,932 mEq/kg (Lawlor et al., 2005). In piglet diets, partially or totally replacing limestone with Ca-formate and adding citric acid reduced the dietary ABC-4 from 308 mEq/kg to 243 mEq/kg, and consequently reduced gastric pH from 4.22 to 3.57 (Huting et al., 2021).

Besides the well-known beneficial effect of a low stomach pH on reducing the incidence of post-weaning diarrhea, a low stomach pH can also positively influence the efficiency of phytase, as most available commercial phytases have an acid pH optimum of activity (pH 3.0 to 5.5; Menezes-Blackburn et al., 2015), in keeping with phytate being highly soluble at low pH. Therefore, replacing limestone with Ca-formate may offset the pH elevation caused by limestone, thereby enhancing phytate degradation. In-vitro data demonstrated that, in the presence of Ca, an acidic pH (2.5) favors phytate hydrolysis, while a greater pH (6.5) reduces phytase activity (Tamim et al., 2004). Data from growing pigs also indicated that the addition of formic acid to diets containing limestone and phytase increased by 7% units the ileal phytate disappearance (Klein et al., 2024), indicating a positive effect of a lower dietary ABC on phytase activity. On the other hand, differences in Ca solubility between limestone and Ca formate may also alter P utilization by the formation of Ca-phytate complexes, as it has been observed in broiler chickens (Walk et al., 2012). To our knowledge, there is no available study testing the effect of using limestone or Ca-formate as main Ca sources in piglet diets on the digestibility of Ca and P, and the efficiency of phytase. Therefore, the objective of this trial was to test the hypothesis that using Ca-formate instead of limestone in piglet diets, reduces the dietary ABC-4, lowers stomach pH, and improves phytase efficiency to degrade phytate and release P.

## Materials and Methods

The study was conducted at the facilities of Schothorst Feed Research (SFR), Lelystad, the Netherlands according to the restrictions of the Animal and Human Welfare Codes in the Netherlands and approved by the Ethics Committee and Institutional Review Board of Schothorst Feed Research (AVD24600202010384, 10 October 2021). Piglets were housed, fed, and managed according to directive 2010/63/EU for the protection of animals used for scientific purposes.

### Animals, housing, and handling

Sixty entire piglets (TN70 × Tempo; Topigs Norsvin, Den Bosch, the Netherlands) were weaned at 29.8 ± 1.10 d [average body weight (BW): 8.83 ± 0.31 kg] and randomly allocated to ten experimental diets and six blocks (replicate) in a randomized complete block design. The trial was performed in two weaning rounds. There were 36 pigs in round 1 and 24 pigs in round 2, for a total of 6 replicate pigs per treatment (based on power calculation). Thus, in round 1, there were four replicates per treatment for treatments 1, 3, 4, 6 to 10, and two replicates per treatment in treatments 2 and 5. In round 2, there were two replicates per treatment for treatments 1, 3, 4, 6 to 10, and four replicates per treatment in treatments 2 and 5. Piglets were housed in pairs in metabolic units (2.0 m × 1.0 m) that were equipped with two feeders, two nipple drinkers, and a slatted floor to allow for the separate collection of feces (partial) and urine (total) samples. Room temperature and relative humidity were mechanically controlled and recorded daily. On day 10 post-weaning, pigs were weighed (average BW = 11.5 ± 1.06 kg) and subsequently separated through the placement of a transparent wall in each metabolic unit, leaving one feeder and one nipple drinker per pig. They were housed individually until day 18 post-weaning (last day of the experiment; average BW = 15.6 ± 1.48 kg). Final pen dimensions at individual housing were 1.0 m × 1.0 m. The health status of the piglets was checked at least once a day by an animal caretaker and more frequently if an animal was found in poor condition. If a piglet was considered unlikely to recover or survive, it was humanely euthanized. In case treatment (antibiotic) was necessary, the pig number, type and duration of treatment, and reason for treatment was recorded. In case of mortality, the piglet BW and cause of death was recorded. Fecal consistency (Guan et al., 2023) was determined daily at a pen level between day 0 and 10 post-weaning and at the individual level between day 10 and 18 post-weaning.

### Diets and feeding

Ten diets based on barley, wheat, corn, and soybean meal were formulated to meet requirements for all essential nutrients except Ca and P following the CVB (2021) recommendations for weaning pigs. Diet formulation followed a 5 × 2 factorial design with 5 dietary treatments and 2 main Ca sources (limestone or Ca-formate; Table 1). The dietary treatments included a positive control (PC) diet without phytase supplementation containing 0.34% standardized total tract digestible (STTD) P and 0.76% Ca, and four negative control (NC) diets with 0.21% STTD P and 0.56% Ca supplemented with phytase at 0, 750, 1,500, and 3,000 FYT/kg (HiPhorius 40; dsm-firmenich, Kaiseraugst, Switzerland), using the analyzed phytase activity of the product (54,740 FYT/g). The PC diet within each Ca source had the same ingredient composition as the NC but contained a greater inclusion rate of monocalcium phosphate (MCP; 1.065% vs. 0.350%, respectively). All diets contained 0.50% of benzoic acid (VevoVitall; dsm-firmenich, Kaiseraugst, Switzerland), 100 g of xylanase/ton feed (Ronozyme WX2000; dsm-firmenich, Kaiseraugst, Switzerland), and 0.50% of TiO_2_ as an indigestible marker (Table 2). The two Ca sources and main feed ingredients (i.e., barley, wheat, soybean meal, wheat middling’s maize, sweet whey powder, and sunflower seed meal) were analyzed for Ca and P by two different labs (NutriControl B.V., Veghel, the Netherlands and MasterLab B.V., Boxmeer, the Netherlands) before diet production to optimize the diet composition. Diets were produced at Research Diet Services (Wijk bij Duurstede, the Netherlands) and were offered to pigs as 3 mm pellets.

**Table 1.** Analyzed Ca content, Ca solubility, and particle size of the calcium sources

Calcium source	Ca, %	Ca solubility, %			Particle size	ABC-4^2^
		5 min	15 min	30 min	GMD^1^, mm	mEq/kg
**Limestone^3^**	40.1	79.33	91.89	100.0	0.237	13,603.8
**Calcium formate**	31.4	94.88	95.74	96.01	0.171	3,141.6

1 GMD, geometric mean diameter.

2 ABC-4, acid binding capacity at pH 4.

3 Limestone brand: minifil-L400 BT (chalk).

**Table 2.** Ingredient and nutrient (calculated and analyzed) composition of the positive control (PC) and negative control (NC) diets containing limestone or Ca formate as the main calcium source

Ca source	Limestone					Calcium formate				
Diet	NC	PC	NC	NC	NC	NC	PC	NC	NC	NC
**Phytase, FYT**	0	0	750	1,500	3,000	0	0	750	1,500	3,000
**Ingredient, %**										
** Barley**	21.19	21.19	21.19	21.19	21.19	21.19	21.19	21.19	21.19	21.19
** Wheat**	17.50	17.50	17.50	17.50	17.50	17.50	17.50	17.50	17.50	17.50
** Corn**	12.50	12.50	12.50	12.50	12.50	12.50	12.50	12.50	12.50	12.50
** Wheat middling’s**	14.00	13.60	14.00	14.00	14.00	13.90	13.50	13.90	13.90	13.90
** Soybean meal, 48% crude protein**	15.00	15.00	15.00	15.00	15.00	15.00	15.00	15.00	15.00	15.00
**Sunflower meal, 37% crude protein**	3.00	3.00	3.00	3.00	3.00	3.00	3.00	3.00	3.00	3.00
** Whey sweet**	7.10	7.10	7.10	7.10	7.10	7.10	7.10	7.10	7.10	7.10
** Soybean oil**	2.30	2.70	2.30	2.30	2.30	2.40	2.80	2.40	2.40	2.40
** Molasses beet**	1.00	1.00	1.00	1.00	1.00	1.00	1.00	1.00	1.00	1.00
** Corn starch**	1.27	0.35	1.27	1.27	1.27	1.02	0.10	1.02	1.02	1.02
** Water**	1.00	1.00	1.00	1.00	1.00	1.00	1.00	1.00	1.00	1.00
** Monocalcium phosphate**	0.35	1.07	0.35	0.35	0.35	0.35	1.07	0.35	0.35	0.35
** Limestone**	0.85	1.05	0.85	0.85	0.85	-	0.15	-	-	-
** Calcium formate**	-	-	-	-	-	1.10	1.15	1.10	1.10	1.10
** Salt**	0.50	0.50	0.50	0.50	0.50	0.50	0.50	0.50	0.50	0.50
** Lys HCl (79%)**	0.48	0.48	0.48	0.48	0.48	0.48	0.48	0.48	0.48	0.48
** Met DL (99%)**	0.16	0.16	0.16	0.16	0.16	0.16	0.16	0.16	0.16	0.16
** Thr L (98%)**	0.17	0.17	0.17	0.17	0.17	0.17	0.17	0.17	0.17	0.17
** Trp L (98%)**	0.04	0.04	0.04	0.04	0.04	0.04	0.04	0.04	0.04	0.04
** Val L (99%)**	0.04	0.04	0.04	0.04	0.04	0.04	0.04	0.04	0.04	0.04
** Vitamin mineral premix^1^**	0.50	0.50	0.50	0.50	0.50	0.50	0.50	0.50	0.50	0.50
** Copper sulphate (99%)**	0.05	0.05	0.05	0.05	0.05	0.05	0.05	0.05	0.05	0.05
** Benzoic acid**	0.50	0.50	0.50	0.50	0.50	0.50	0.50	0.50	0.50	0.50
** Xylanase**	0.01	0.01	0.01	0.01	0.01	0.01	0.01	0.01	0.01	0.01
** Titanium dioxide**	0.50	0.50	0.50	0.50	0.50	0.50	0.50	0.50	0.50	0.50
** Phytase, mg/kg^2^**	-	-	13.70	27.40	54.80	-	-	13.70	27.40	54.80
**Calculated values, %**										
** Net energy, kcal/kg**	2,275	2,275	2,275	2,275	2,275	2,275	2,275	2,275	2,275	2,275
** Digestible Lys^3^**	1.04	1.04	1.04	1.04	1.04	1.04	1.04	1.04	1.04	1.04
** Digestible P^4^**	0.21	0.34	0.21	0.21	0.21	0.21	0.34	0.21	0.21	0.21
** Total P**	0.47	0.63	0.47	0.47	0.47	0.47	0.63	0.47	0.47	0.47
** Total Ca**	0.56	0.76	0.56	0.56	0.56	0.56	0.76	0.56	0.56	0.56
** ABC-4,^5^ mEq/kg**	326	351	326	326	326	197	213	197	197	197
**Analyzed values, %**										
** Dry matter**	87.8	88.2	88.3	88.2	88.2	88.1	88.2	88.2	88.1	88.1
** Ash**	5.17	5.87	5.27	5.21	5.27	5.25	5.86	5.20	5.21	5.23
** Crude protein**	17.0	16.8	17.0	17.0	17.2	17.0	17.0	17.1	17.1	17.1
** Crude fat (AH)^6^**	4.54	4.95	4.61	4.61	4.60	4.64	4.98	4.62	4.63	4.66
** Starch**	34.2	33.5	34.1	34.5	34.0	33.6	32.9	34.1	34.3	34.4
** Sugar**	8.70	8.70	9.10	8.60	8.60	8.70	8.50	8.40	8.60	9.30
** Non-phytate P**	0.22	0.37	0.21	0.21	0.22	0.22	0.38	0.21	0.22	0.23
** Phytate P^7^**	0.24	0.25	0.27	0.27	0.25	0.26	0.27	0.27	0.26	0.25
** Total Ca**	0.59	0.76	0.55	0.54	0.52	0.54	0.73	0.53	0.54	0.54
** Total P**	0.46	0.62	0.48	0.48	0.47	0.48	0.65	0.48	0.48	0.48
** Ti**	0.30	0.30	0.31	0.30	0.30	0.30	0.30	0.30	0.30	0.30
** ABC-4, mEq/kg**	247	278	255	235	256	222	233	224	227	229
** Phytase, FYT/kg**	254	278	840	1,386	2,439	298	345	760	1,416	2,376

1 Provided per kg of diet (0.50% inclusion): vitamin A (retinyl acetate, 10,000 IU), vitamin D_3_ (cholecalciferol, 2,000 IU), vitamin E (dl-α-tocopherol, 40 mg), vitamin K_3_ (menadione, 1.5 mg), vitamin B_1_ (thiamine, 1.0 mg), vitamin B_2_ (riboflavin, 4.0 mg), vitamin B_6_ (pyridoxin-HCl, 1.5 mg), vitamin B_12_ (cyanocobalamin, 0.020 mg), niacin (30 mg), D-pantothenic acid (15 mg), choline chloride (150 mg), folic acid (0.4 mg), biotin (0.05 mg), Fe (FeSO_4_.H_2_O, 100 mg), Cu (CuSO_4_.5H_2_O, 20 mg), Mn (MnO, 30 mg), Zn (ZnSO_4_.H_2_O, 70 mg), I (KI, 0.7 mg), Se (Na_2_SeO_3_, 0.25 mg).

2 Dosing of phytase (in mg/kg diet) was based on the analyzed phytase activity (54,740 FYT/g) of the product.

3 Expressed as standardized ileal digestible Lys.

4 Expressed as standardized total tract digestible P.

5 ABC-4, Acid binding capacity at pH 4.

6 Crude fat analysis after acid hydrolysis.

7 Phytate P was calculated by subtracting the non-phytate P from the total P.

Pigs were allowed *ad libitum* access to feed from day 0 to 10 post-weaning, and were changed to a semi-*ad libitum* feeding schedule (twice a day at a feeding level of 3.2 × metabolic BW) from days 10 to 16 post-weaning. From day 16 onwards, the semi-*ad libitum* feeding schedule consisted of 6 equal meals a day to reach a steady flow of digesta through the digestive tract. On the last day of the experiment (day 18), piglets were fed at 6 h before sacrifice (1/6 of the daily portion), 3.5 h before sacrifice (1/6 of the daily portion), and 1 h before sacrifice (1/4 of the daily portion), to ensure that all parts of the digestive system were filled with sufficient content. Feed intake was recorded at a pen level from day 0 to 10 post-weaning and at individual level from day 10 post-weaning onwards.

### Sample collection and bone measurements

Diet samples were collected before and after pelleting at the feed mill at different time points during passage or bagging, respectively. On the weaning day (day 0 of the experiment) and day 7 post-weaning, blood samples were collected by jugular venipuncture into plasma (heparinized) and serum tubes, centrifuged, and stored at 4 °C in 1 mL tubes for further analyses. During the last 5 days of the experiment (day 13 to 18 post-weaning), fecal samples were collected twice a day (830 and 1,500 h) from the individual pens. Urine was collected into a bucket from a funnel located in the tray underneath the pen. Hydrochloric acid was added to the urine after each sampling time (5 mL/time; adjusted to the actual volume of urine that was collected). Fecal and urine samples were stored at 4 °C during the collection period, and at −20 °C during storage before freeze-drying. On day 18, piglets were humanely euthanized via intracardiac injection with T61 (MSD Animal Health, Boxmeer, the Netherlands) after sedation with Zoletil (Virbac, Barneveld, the Netherlands), and a third blood sample and the left front foot were immediately collected. The abdominal cavity was subsequently opened, clamps were placed proximal and distal to the stomach and small intestine to prevent the movement of digesta, and the entire gastrointestinal tract was carefully removed and placed on a table. The stomach was separated from the intestinal tract, its content was emptied into a bucket, homogenized by manual mixing, and pH was immediately measured with a portable pH meter (Mettler-Toledo B.V., Tiel, the Netherlands). The small intestine was divided into 3 equal sections, the last 2 m from the second third of the small intestine was considered the jejunum, and the distal 2 m from the small intestine was considered the terminal ileum. The mentioned segments were dissected to collect its contents by gentle stripping. Digesta samples were stored at −20 °C before freeze-drying (Lagos and Stein, 2019).

The collected feet were autoclaved at 120 °C for 1 h and the third and fourth metacarpals were collected and cleaned of residual flesh. Metacarpals were first dried for 24 h at 40 °C followed by another 24 h of drying at 70 °C. Bones were soaked in petroleum ether for 48 h under a chemical hood to remove marrow and fat. After letting the bones dry overnight in the hood, they were dried for 4 h at 103 °C, allowed to cool down for 30 min in a desiccator, and weighed (fat-free weight). Bones were then ashed at 800 °C for 18 h, allowed to cool down for 1 h in a desiccator, and weighed again (ash weight). Bone ash was calculated by dividing the ash weight by the fat-free weight, and expressed as a percentage.

### Sample analysis

Ingredient, diet, fecal, and digesta samples were ground to 1 mm before chemical analysis at the laboratory of Schothorst Feed Research. All chemical analysis were performed in duplicate. All diets were analyzed for dry matter (DM; NEN-ISO-6496, 1999), ash (NEN-ISO-5984, 2003), crude protein (N × 6.25; NEN-EN-ISO-16634, 2004), crude fat after acid hydrolysis (NEN-EN-ISO-11085, 2015), starch (NEN-EN-ISO-15914, 2005), and sugar (NEN-EN-ISO/IEC-17025, 2018). Limestone and Ca-formate were analyzed for Ca solubility using an in vitro solubility method (Kim et al., 2019) and for particle size according to the procedure suggested by the American Society of Agricultural Engineers (ASAE, 2003). The two Ca sources and all diets were also analyzed for ABC-4 (Lawlor et al., 2005). All diets and digesta samples were analyzed for free P (not bound to P) as P-vanadate-molybdate by colorimetry at 430 nm after HCl extraction followed by deproteination using trichloroacetic acid and the addition of vanadate-molybdate (in-house method; Schothorst Feed Research, Lelystad, the Netherlands). Ingredient, diet, fecal, digesta, urine, and serum samples were analyzed for P by ultraviolet visible spectrophotometry (NEN-ISO-6491, 1999). Ingredient, diet, fecal, ileal digesta, and serum samples were analyzed for Ca by atomic absorption spectrometry (NEN-EN-ISO-6869, 2001). Calcium was not analyzed in the jejunal samples due to the limited sample size collected. Fecal samples were analyzed for dry matter and ash content. Plasma samples were prepared as described by Frieler et al. (2009) and analyzed by UPLC/MS according to the method of Leung et al. (2011). Diet, fecal, and digesta samples were analyzed for Ti following the procedure described by Short et al. (1996). Phytase activity was analyzed by colorimetry at Biopract GmbH, Berlin, Germany, in the phytase concentrate and all pellet diets (EN-ISO-30024, 2024).

### Calculations and statistical analyses

The sample size calculation was performed using the ASAMPLESIZE procedure in GenStat for Windows Version 22 (VSN International Ltd, Hemel Hempstead, UK) with a significant level of α < 0.05 and a power of 0.80. Response parameters were apparent total tract digestibility (ATTD) of Ca and plasma inositol. Organic matter was calculated by subtracting the ash content from the DM content in diet and fecal samples, and phytate P (PP) was calculated by subtracting the non-phytate P from the total P content in diet and digesta samples. The dietary apparent jejunal digestibility (AJD) of P, apparent ileal digestibility (AID) of PP, P, and Ca, and ATTD of Ca and P were calculated using the index method with TiO_2_ as index following the equation described by Kong and Adeola (2014). The concentration of AID and apparent total tract digestible (ATTD) P and Ca was calculated by multiplying the coefficient of AID and ATTD of P and Ca by the dietary P and Ca content, respectively. Phosphorus retention (in g/kg and %) was calculated following the methodology described by Huting et al. (2023).

All data were screened for outliers, which were identified as the standardized residuals that exceeded 2.5 times the standard error of the data set. Outliers were dropped from analysis. For the digestibility data, an extra quality check was performed following the protocol for digestibility studies of CVB (2005). If the dietary ATTD of organic matter (data not shown) of an animal differed by more than 2.5 times the SD from the average, the piglet was considered an outlier. Data were subjected to analysis of variance (ANOVA) by GenStat.

For the digestibility, digestible concentration, retention, stomach pH, and bone data, the model included the main effects of Ca source and dietary treatment and the interaction between main effects, and the random effect of replicate. Data for blood metabolites obtained on day 0 were used as a covariate to analyze blood data from days 7 to 18. Repeated measures were used to analyze the effect of time (day) on the plasma inositol and serum Ca and P of piglets. The model included the main effects of Ca source, dietary treatment, and day and the different interactions among or between main effects. The time effect was day, and the random effect was replicate. Feed intake was recorded individually after day 10 post-weaning, therefore, for all response parameters, the experimental unit was the piglet. Treatment means were compared using the Least Significant Differences (Fisher’s LSD method). A T-probability of *P *≤ 0.05 was considered statistically significant, while 0.05 < *P *≤ 0.10 indicated a tendency. Regression and curve fitting analyses were performed with the four NC diets of each Ca source to determine the equivalence in phytase units. Thus, an exponential regression model as shown below was fitted to the dietary concentration of ATTD Ca and P,


$$Y=A+{B\times (R}^{X})+\text{residual}\;\text{error}$$

where *Y* is the response variable (AID or ATTD Ca and P), *A* is the upper asymptote value, *B* is the response compared to upper asymptote without phytase supplementation, *R* is the nonlinear slope parameter, and *X* is the phytase dose level (calculated phytase activity). Because the concentration of AID and ATTD Ca and P had the same response pattern, only results for ATTD Ca and P are shown. To evaluate phytase efficiency across Ca sources, 99% of the maximum response (variable *A* from the model) from the lower-performing diet (limestone or Ca-formate) was used as reference. This value reflects the asymptotic nature of the model, providing a practical threshold that is close enough to the theoretical upper limit for meaningful comparison.

## Results

**Figure 1.**
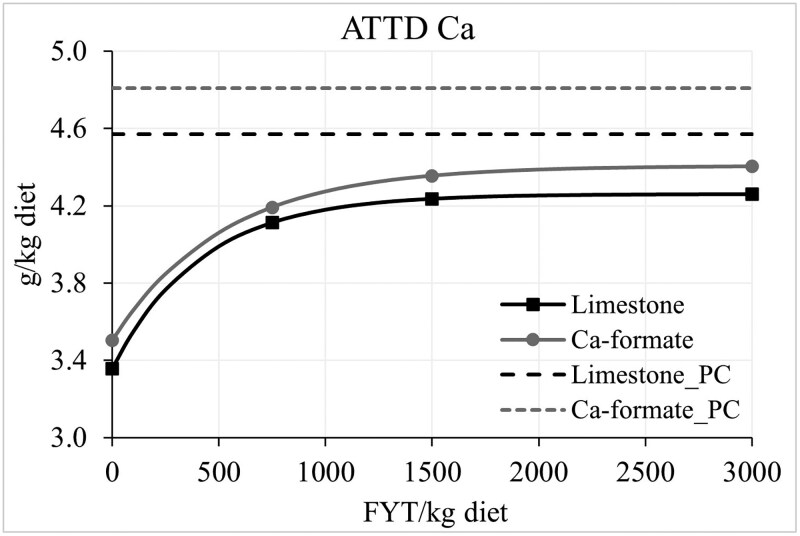
Observed data points and fitted exponential model (solid curve) of the average concentration of ATTD Ca in diets containing limestone [*Y* = 4.26 + (−0.90 × 0.998 X); *R*^2^ = 68.2; *P* < 0.01] or Ca-formate [*Y* = 4.41 + (−0.90 × 0.998 X); *R*^2^ = 66.3; *P* < 0.01] as a function of phytase inclusion level. The maximum response for the limestone diets was 4.26 g/kg (SE = 0.087), the coefficient that escalates the exponential term was −0.903 (SE = 0.314), and the growth rate was 0.998 (SE = 0.00122). The maximum response for Ca-formate diets was 4.41 g/kg (SE = 0.097), the coefficient that escalates the exponential term was −0.903 (SE = 0.141), and the growth rate was 0.998 (SE = 0.00087). The dashed lines represent the average concentration of ATTD Ca in the positive control (PC) diets containing limestone or Ca-formate.

**Figure 2.**
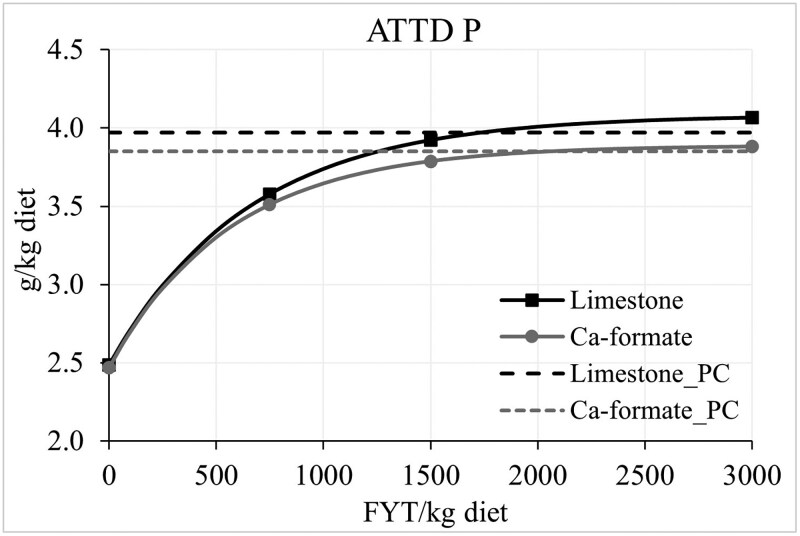
Observed data points and fitted exponential model (solid curve) of the average concentration of ATTD P in diets containing limestone [*Y* = 4.08 + (−1.60 × 0.998 X); *R*^2^ = 95.5; *P* < 0.01] or Ca-formate [*Y* = 3.89 + (−1.42 × 0.998 X); *R*^2^ = 92.9; *P* < 0.01] as a function of phytase inclusion level. The maximum response for the limestone diets was 4.08 g/kg (SE = 0.058), the coefficient that escalates the exponential term was −1.60 (SE = 0.078), and the growth rate was 0.998 (SE = 0.0002). The maximum response for Ca-formate diets was 3.89 g/kg (SE = 0.062), the coefficient that escalates the exponential term was −1.42 (SE = 0.088), and the growth rate was 0.998 (SE = 0.00031). The dashed lines represent the average concentration of ATTD P in the positive control (PC) diets containing limestone or Ca-formate.

In round 1, three pigs (from treatments 1, 3, and 4) had to be treated with antibiotics before or during the collection period due to poor health conditions and were removed from the study. Therefore, in round 2, three more pigs entered the study to reach 6 replicates per treatment. An interaction (*P *< 0.05) between Ca source and dietary treatment was observed for stomach pH and ADJ of PP and P. No difference in piglet stomach pH was observed between Ca sources for any of the NC diets, but for the PC diet, using Ca-formate instead of limestone resulted in lower stomach pH (Table 3). The AJD of PP was not different between Ca sources for the NC without and with phytase at 1,500 and 3,000 FYT, but for the NC + 750 FYT diet, the ADJ of PP was greater in the Ca-formate diet than in the limestone diet (*P *< 0.05). For the PC diet, the Ca-formate diet resulted in lower ADJ of PP than the limestone diet (*P *< 0.05). There was no effect of Ca source on the AJD of P in the PC diet or the NC diet without or with phytase at 1,500 and 3,000 FYT, but the NC + 750 FYT had greater (*P *< 0.05) AJD of P if Ca-formate instead of limestone was used as the main Ca source. For the coefficient (%) and concentration (g/kg diet) of AID and ATTD of minerals, no interaction was observed. However, regardless of the dietary treatment, the coefficient and concentration of AID and ATTD Ca were greater (% and g/kg diet; *P *< 0.05), whereas the coefficient and concentration of AID and ATTD P were lower (% and g/kg diet; *P *< 0.05) in the Ca-formate diets than in the limestone diets. Regardless of the Ca source, there was no difference in the AID of PP between the NC and the PC, but the AID of PP in the NC + 750 FYT was greater (*P *< 0.05) than in the control diets, but lower (*P *< 0.05) than in the NC + 1,500 or 3,000 FYT. The AID and ATTD of P was greater (*P *< 0.05) in the PC than in the NC diet, and it increased (*P *< 0.05) stepwise with increasing phytase dose up to 1,500 FYT. There was no difference in the AID and ATTD of Ca between the NC and PC diets, but phytase supplementation increased (*P *< 0.05) the response up to 1,500 FYT. Regardless of the Ca source, the concentration of AID and ATTD P and Ca was greater in the PC and the phytase-supplemented diets than in the NC diet (*P *< 0.05). The concentration of AID P was greater (*P *< 0.05) in the NC diets supplemented with 1,500 or 3,000 FYT than in the NC + 500 FYT or PC diet, with no significant difference between the latter two. The concentration of ATTD P was greater (*P *< 0.05) in the NC + 1,500 or 3,000 FYT than in the NC + 500 FYT, but no different from the PC diet. The concentration of ATTD Ca was greater (*P *< 0.05) in the NC + 1,500 FYT than in the NC + 500 FYT diet, with the NC + 3,000 FYT diet having intermediate values. However, the ATTD Ca in the phytase-supplemented diets was lower (*P *< 0.05) than in the PC diet. The concentration of ATTD Ca in the limestone and Ca-formate diets increased exponentially (*P *< 0.05) with increasing dietary phytase (Figure 1). To reach 99% of the maximum response for ATTD Ca in limestone diets (4.26 g/kg; i.e., variable A from the regression model), 1,259 FYT/kg of phytase are needed in the limestone diets while only 992 FYT/kg of phytase are needed in the Ca-formate diets. The maximum response for Ca-formate diets was 4.41 g/kg ATTD Ca. The concentration of ATTD P in the limestone and Ca-formate diets increased exponentially (*P *< 0.05) with increasing dietary phytase (Figure 2). To reach 99% of the maximum response for Ca-formate diets (ATTD *P* = 3.89 g/kg), 1,944 FYT/kg of phytase are needed in the Ca-formate diets, but only 1,232 FYT/kg of phytase are needed in the limestone diets. The maximum response for limestone diets was 4.08 g/kg ATTD P. In the limestone diets, 1,727 FYT are needed to reached the concentration of ATTD P in the corresponding PC diet (4.00 g/kg), whereas in the Ca-formate diets, 2,052 FYT are needed to reach the ATTD P in the corresponding PC diet (3.82 g/kg). Data for the coefficient of ATTD of Ca and P is available in Supplementary Data.

**Table 3.** Effect of Ca source and dietary treatment on the gastric pH, apparent jejunal digestibility (AJD, %), apparent ileal digestibility (AID, %), and apparent total tract digestibility (ATTD, %) of phytate P (PP), P, and Ca, and the concentration of apparent ileal digestible (AID, g/kg) and apparent total tract digestible (ATTD, g/kg) P and Ca in diets fed to weaned piglets^1^

Ca source	Diet	Gastric	AJD of, %		AID of,%			ATTD of, %		AID, g/kg		ATTD, g/kg	
	Treatment^2^	pH	PP	P	PP	P	Ca	P	Ca	P	Ca	P	Ca
**Limestone**	PC	4.57^b^	31.7^b,c^	56.6^b,c^	13.5	51.6	59.5	60.2	60.1	3.40	4.53	3.97	4.57
	NC	4.39^b^	21.8^a,b^	45.7^a^	11.4	41.6	58.8	50.0	62.4	2.07	3.17	2.49	3.36
	NC + 750	3.80^a,b^	41.6^c^	48.6^a^	65.4	67.1	79.1	73.2	75.8	3.34	4.26	3.64	4.08
	NC + 1,500	4.06^a,b^	64.0^d^	66.4^d^	77.3	79.2	85.2	79.0	80.5	3.94	4.59	3.93	4.34
	NC + 3,000	4.32^b^	71.1^d^	72.3^d^	86.0	80.5	85.0	81.7	78.3	4.00	4.17	4.06	4.21
**Ca-formate**	PC	3.41^a^	13.7^a^	51.7^a,b^	4.3	52.4	64.8	58.3	65.9	3.45	4.76	3.85	4.81
	NC	4.38^b^	25.6^a,b^	47.3^a^	9.8	39.1	70.1	49.6	65.9	1.95	3.73	2.47	3.51
	NC + 750	4.36^b^	61.1^d^	64.5 ^c,d^	55.1	65.2	84.1	70.2	78.6	3.25	4.48	3.50	4.18
	NC + 1,500	4.25^b^	64.0^d^	67.2^d^	73.8	74.5	89.8	76.6	82.4	3.71	4.78	3.82	4.38
	NC + 3,000	4.44^b^	68.7^d^	67.5^d^	73.0	73.0	87.3	77.6	82.4	3.63	4.69	3.87	4.39
	SEM	0.788	4.76	2.77	3.65	2.11	2.61	1.08	1.87	0.110	0.168	0.056	0.106
**Limestone**		4.23	46.1	57.9	50.7^b^	64.0^b^	73.5^a^	68.8^b^	71.4^a^	3.35^b^	4.14^a^	3.62^b^	4.11^a^
**Ca-formate**		4.17	46.6	59.7	43.2a	60.8^a^	79.2^b^	66.4^a^	75.0^b^	3.20^a^	4.49^b^	3.50^a^	4.25^b^
**SEM**		0.125	2.13	1.24	1.63	0.94	1.17	0.48	0.84	0.049	0.075	0.025	0.047
	PC	3.99	22.7	54.2	8.9^a^	52.0^b^	62.2^a^	59.3^b^	63.0^a^	3.43^b^	4.64^b^	3.91^c^	4.69^d^
	NC	4.39	23.7	46.5	10.6^a^	40.4^a^	64.5^a^	49.8^a^	64.1^a^	2.01^a^	3.45^a^	2.48^a^	3.43^a^
	NC + 750	4.08	51.4	56.6	60.2^b^	66.2^c^	81.6^b^	71.7^c^	77.2^b^	3.30^b^	4.37^b^	3.57^b^	4.13^b^
	NC + 1,500	4.16	64.0	66.8	75.6^c^	76.9^d^	87.5^c^	77.8^d^	81.5^c^	3.83^c^	4.68^b^	3.87^c^	4.36^c^
	NC + 3,000	4.38	69.9	69.9	79.5^c^	76.7^d^	86.1^b,c^	79.6^d^	80.4^b,c^	3.82^c^	4.43^b^	3.96^c^	4.30^b,c^
	SEM	0.195	3.37	1.96	2.58	1.49	1.84	0.77	1.33	0.078	0.119	0.039	0.075
***P*-value**													
** NC vc PC**													
** Ca source × Dietary treatment**		0.04	0.01	<0.01	0.49	0.37	0.51	0.54	0.87	0.40	0.68	0.61	0.92
** Ca source**		0.78	0.86	0.33	<0.01	0.02	<0.01	<0.01	<0.01	0.03	<0.01	<0.01	<0.01
** Dietary treatment**		0.50	<0.01	<0.01	<0.01	<0.01	<0.01	<0.01	<0.01	<0.01	<0.01	<0.01	<0.01

a-c Means within a column lacking a common superscript letter are different (*P *< 0.05).

1 Data for the interaction are least squares means of 5 or 6 observations.

2 PC, positive control with normal Ca and P level; NC, negative control with reduced Ca and P level; NC + 750, 1,500, 3,000, Phytase units are expressed as FYT/kg feed.

There was an interaction (*P *< 0.05) between Ca source and dietary treatment levels for urinary P (g). In the PC and NC + 3,000 diets, the amount of P in urine was greater if diets contained limestone than if diets contained Ca-formate, with no effect of Ca source in the other three NC diets (Table 4). A tendency (*P *< 0.10) for an interaction was observed for retained P (g), which followed the same trend as urinary P (g) in the PC diets. Regardless of the dietary treatment, a tendency (*P *< 0.10) for a greater digestible P (g) and P retention (%) was observed in the limestone diets compared to the Ca-formate diets. Likewise, regardless of the Ca source, the dietary treatment influenced the piglet P intake (g), digestible P (g), retained P (g), P retention (%), and bone ash (%). Phosphorus intake of piglets was greater (*P *< 0.05) in the PC diet than in the four NC diets. The amount of digestible P was the lowest (*P *< 0.05) in the NC diet, lower (*P *< 0.05) in the NC + 750 than in the two high phytase diets, and lower in the PC than in the NC + 3,000 diet. The amount of retainable P was the lowest (*P *< 0.05) in the NC diet, lower (*P *< 0.05) in the NC + 750 diet than in the two high phytase diets, but the PC diet was not significantly different from the phytase-supplemented diets. Phosphorus retention by piglets was the lowest (*P *< 0.05) in the NC diet, lower (*P *< 0.05) in the PC diet than in the phytase-supplemented diets, and lower (*P *< 0.05) in the NC + 750 diet than in the two high phytase diets. The concentration of bone ash of piglets was greater (*P *< 0.05) in the PC and the phytase supplemented diets than in the NC diet.

**Table 4.** Effect of Ca source and dietary treatment on P intake (Int.), digestible P (DigP), urinary (Uri.) P, retained P (RetP), P retention (Ret.), and bone ash of weaned piglets^1^

Ca-source	Diet treatment^2^	P Int., g	DigP, g	Uri. P, g	RetP, g	P Ret., %	Bone ash, %
**Limestone**	PC	23.6	14.3	1.30^e^	14.1^y,z^	57.8	40.8
	NC	17.3	8.7	0.03^a^	8.7^v^	50.1	36.4
	NC + 750	16.2	12.6	0.5^b,c,d^	11.9^w^	69.0	41.9
	NC + 1,500	19.1	15.1	0.78 ^c,d^	14.3^z^	74.8	40.8
	NC + 3,000	18.6	15.3	1.22^e^	14.1^y,z^	75.6	43.1
**Ca-formate**	PC	21.7	12.6	0.43^b,c^	12.2^w,x^	56.3	41.3
	NC	18.3	9.0	0.01^a^	9.0^v^	49.4	37.9
	NC + 750	18.6	13.0	0.25^a,b^	12.8^w,x,y^	68.7	41.6
	NC + 1,500	17.4	13.3	0.45^b,c^	12.9^w,x,y,z^	74.0	39.8
	NC + 3,000	18.7	14.5	0.84^d^	13.6^x,y,z^	73.1	40.6
	SEM	0.91	0.59	0.131	0.51	1.19	1.36
**Limestone**		19.0	13.2^y^	0.77^b^	12.6	65.5^y^	40.6
**Ca-formate**		18.9	12.5^x^	0.39^a^	12.1	64.3^x^	40.2
**SEM**		0.41	0.27	0.059	0.23	0.54	0.62
	PC	22.6^b^	13.5^b,c^	0.86	13.1^b,c^	57.0^b^	41.0^b^
	NC	17.8^a^	8.9^a^	0.02	8.9^a^	49.8^a^	37.2^a^
	NC + 750	17.4^a^	12.8^b^	0.38	12.4^b^	68.9^c^	41.7^b^
	NC + 1,500	18.3^a^	14.2 ^c,d^	0.61	13.6^c^	74.4^d^	40.3^b^
	NC + 3,000	18.6^a^	14.9^d^	1.03	13.8^c^	74.3^d^	41.8^b^
	SEM	0.64	0.42	0.093	0.36	0.84	0.96
***P*-value**							
** Ca source × Dietary treatment**		0.11	0.19	0.03	0.05	0.89	0.65
** Ca source**		0.91	0.10	< 0.01	0.16	0.09	0.72
** Dietary treatment**		< 0.01	< 0.01	< 0.01	< 0.01	< 0.01	0.01

a-e Means within a column lacking a common superscript letter are different (*P *< 0.05).

v-z Means within a column lacking a common superscript letter tend to be different (*P *< 0.10).

1 Data for the interaction are least squares means of 5 or 6 observations.

2 PC, positive control with normal Ca and P level; NC, negative control with reduced Ca and P level; NC + 750, 1,500, 3,000, Phytase units are expressed as FYT/kg feed.

**Figure 3.**
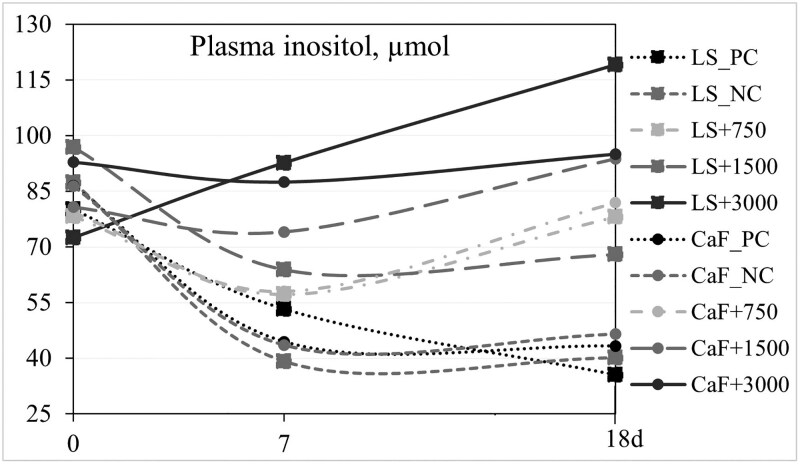
Plasma inositol of pigs fed 5 dietary treatments [PC= positive control, NC= negative control, and negative control +750, +1,500, and +3,000 FYT phytase/kg feed] containing limestone (LS) or Ca-formate (CaF) as main Ca source during days 0 to 18.

Blood metabolites were analyzed at different time points (days 0, 7, and 18), using day zero as a baseline. The three-way interaction (sampling day × Ca source × dietary treatment) was not significant as well as the 2 two-way interactions: Ca source × dietary treatment and Ca source × day, therefore, these data are not shown. There was a tendency (*P *< 0.10) for an interaction between day and dietary treatment for Ca in serum of piglets because at day 18, there was no effect of dietary treatment on serum Ca, but on day 7, piglets fed the PC diet had greater Ca in serum than piglets fed the NC + 1,500 diet (Table 5). An interaction (*P *< 0.05) between day and dietary treatment was observed for plasma inositol of pigs as there was no effect of day on the concentration of inositol in plasma in the PC and NC diets, but in the phytase supplemented diets, plasma inositol was greater at day 18 than at day 7. Regardless of Ca source and day, piglet serum P was the lowest (*P *< 0.05) in the NC diet and greater (*P *< 0.05) in the NC + 3,000 diet than in the NC + 1,500 diet. Plasma inositol in the NC + 750 and NC + 1,500 diets was greater (*P *< 0.05) than in the NC and PC diet, but lower (*P *< 0.05) than in the NC + 3,000 diet. Regardless of Ca source and dietary treatment, serum Ca and plasma inositol of pigs were greater at day 18 than at day 7. Data for plasma inositol of piglets at days 0, 7, and 18 are presented in Figure 3.

**Table 5.** Effect of Ca source, dietary treatment, and day of sampling on serum Ca and P and plasma inositol of weaned piglets^1^^,^^2^

Ca source	Diet	Day of	Serum, mg/L		Plasma, µmol
	treatment^3^	sampling	Ca^4^	P	Inositol
	PC	D7	114.9^y^	90.1	48.1^a,b^
	NC		107.5^x,y^	73.9	41.8^a^
	NC + 750		108.2^x,y^	85.2	58.2^b,c^
	NC + 1,500		104.9^x^	85.0	70.0 ^c,d^
	NC + 3,000		107.8^x,y^	91.6	89.5^e^
	PC	D18	105.0^x^	90.5	39.5^a^
	NC		111.7^x,y^	73.5	42.9^a^
	NC + 750		106.7^x^	93.0	80.7^d,e^
	NC + 1,500		108.1^x,y^	90.2	85.4^e^
	NC + 3,000		105.0^x^	94.7	106.4^f^
	SEM		2.78	2.34	4.93
**Limestone**			106.5^x^	87.9	65.3
**Ca-formate**			109.5^x^	85.7	67.3
**SEM**			1.26	1.07	2.23
	PC		110.0	90.3^b,c^	43.8^a^
	NC		109.6	73.7^a^	42.4^a^
	NC + 750		107.5	89.1^b,c^	69.4^b^
	NC + 1,500		106.5	87.6^b^	77.8^b^
	NC + 3,000		106.4	93.2^c^	97.9^c^
	SEM		1.97	1.65	3.49
		D7	108.7	85.2^a^	61.6^a^
		D18	107.3	88.4^b^	71.0^b^
		SEM	1.24	1.04	2.20
***P*-value**					
** Dietary treatment × Day**			0.09	0.38	0.01
** Ca source**			0.09	0.17	0.62
** Dietary treatment**			0.56	< 0.01	< 0.01
** Day**			0.45	0.03	0.003

a-e Means within a column lacking a common superscript letter are different (*P *< 0.05).

1 Data for the two-way interaction are least squares means of 10 or 12 observations.

2 The three-way interaction (Ca source × Dietary treatment × Day) and the two-way interactions Ca source × Dietary treatment and Ca source × Day for blood metabolites were not significant (*P* < 0.10). Therefore, the data is not shown.

3 PC, positive control with normal Ca and P level; NC, negative control with reduced Ca and P level; NC + 750, 1,500, 3,000, Phytase units are expressed as FYT/kg feed.

4 Eventhought the *P*-value indicates a tendency for a Ca source effect, the Fisher’s LSD method did not detect differences between the two treatments.

## Discussion

The analyzed values for Ca and P in the experimental diets were in close agreement (± 0.04%) with calculated values because diets were reoptimized using the analyzed values for Ca and P in feed ingredients. Likewise, the analyzed values for dietary ABC-4 were in line with calculated values, although the analyzed ABC-4 in the limestone and Ca-formate diets were, on average, 77 mEq/kg lower and 27 mEq/kg greater than calculated values, respectively. This is because diets were not reoptimized using the analyzed ABC-4 values in the Ca sources, which were greater for limestone (13,604 vs. 18,000 mEq/kg) and lower for Ca-formate (3,142 vs. 2,150 mEq/kg) than referenced values. Nevertheless, these data support previous results demonstrating the additivity of ABC-4 in complete diets (Lawlor et al., 2005; Batonon-Alavo et al., 2016). Phytase recovery in pellet diets was within acceptable levels (79% to 112% of expected values), as the inclusion of phytase in diets was based on analyzed instead of expected phytase activity.

The observation that the use of Ca-formate to replace limestone in piglet diets had a limited effect on gastric pH was not expected and is in contrast with the finding of a reduction in gastric pH of piglets when limestone in diets was partially or totally replaced by Ca-formate (Huting et al., 2021). This might be a consequence of the relatively low ABC-4 value of the limestone used in the present trial compared to published data on limestone (18,384 ± 769.7 mEq/kg; Stas et al., 2022), although the average gastric pH value obtained in this trial was the same as that reported by Huting et al. (2021) in pigs fed limestone-containing diets (e.g., 4.2). It is possible that feed restriction (i.e., using a semi-*ad libitum* feeding schema) might have played a role in these results and that a different outcome would have been observed if piglets were fed ad libitum, but this proposition needs validation. Nevertheless, data from this trial demonstrate that using Ca-formate instead of limestone reduces the dietary ABC-4, but reject our hypothesis that this further results in lower gastric pH.

A low pH environment favors phytase activity because the majority of commercial phytases are acidic, and phytate solubility is maximized at a low pH (Maenz, 2001). Thus, a potential reduction in gastric pH by using Ca-formate as a main Ca source was expected to increase phytate degradation by phytase and P digestibility. Previous data demonstrated a synergistic effect between phytase and organic acids on the digestibility of P in weanling and growing pigs (Jongbloed et al., 2000; Omogbenigun et al., 2003; Blank et al., 2012). However, the AID of PP and the AID and ATTD of P obtained in the present trial indicated the opposite; using Ca-formate as the primary Ca source in piglet diets negatively affected phytate breakdown and P digestibility. On the contrary, the AID and ATTD of Ca results indicated that replacing limestone with Ca-formate increases the digestibility of Ca in piglet diets. The lack of interaction between Ca source and dietary treatment for the AID and ATTD of Ca and P indicated that the observed effect of Ca source on the dietary Ca and P digestibility is independent of the dietary treatment. These results, along with the lack of a Ca source effect on gastric pH suggest that the Ca solubility of the Ca source plays a more important role on mineral digestibility than its buffering capacity. The lower P digestibility observed in piglets fed Ca-formate compared with limestone diets may be associated with the high solubility of Ca-formate, which results in more Ca available to bind free P and thus, the formation of undissolved Ca-P complexes in the small intestine (Stein et al., 2011). Similar results were observed in broiler chickens when highly soluble Ca sources were used in replacement of limestone (Walk et al., 2012). These outcomes underline the importance of avoiding excess Ca in pig diets to prevent compromising P digestibility and animal performance, especially when using calcium sources other than limestone.

Studies with pigs have shown no difference between the coefficients of AID and ATTD of Ca and P (Dilger and Adeola, 2006; Zhang et al., 2016). Although the present trial did not evaluate the effect of collection site on the digestibility of Ca and P, data indicated that the ATTD of P is, on average, 5% units greater than the AID. In contrast, the ATTD of Ca is about 3% units lower than the AID. These findings are consistent with recent publications, including a meta-analysis showing that, in pigs, the ATTD is significantly greater than the AID of P (Park and Kim, 2025). Likewise, a study using growing pigs suggested that a small amount of endogenous Ca is secreted into the hindgut, as the ATTD of Ca was found to be 4% units greater than the AID (Lagos et al., 2023). Additionally, data from the present trial demonstrate that by the end of the jejunum, an substantial proportion of P has been absorbed, as indicated by the already high AJD, which is on average 4% units greater than the AID.

The exponential model for the concentration of ATTD P indicated that the efficiency of phytase is compromised when Ca-formate replaces limestone in piglet diets as more phytase (nearly 750 FYT) is needed to reach the same dietary concentration of ATTD P. Similarly, the phytase equivalence to the corresponding PC indicated that a greater inclusion of phytase (325 FYT) is needed in diets containing Ca-formate compared to those with limestone. This fully rejects the hypothesis that the efficiency of phytase is improved when Ca-formate is used as the primary Ca source for piglets. Moreover, the model also indicated that the reduction in the concentration of dietary ATTD P by Ca-formate is specific to the phytase diets, suggesting that the available Ca creates complexes with phytate, compromising the efficiency of phytase in degrading phytate (González-Vega and Stein, 2014). The released P may be further trapped in undigestible Ca-P complexes, which would explain the widening difference in the concentration of ATTD P between the Ca-formate and limestone diets with increasing phytase doses in diets, as well as the difference in ATTD P concentration between the PC diets, with a greater value in the limestone diet compared to the Ca-formate diet. Likewise, the observation that Ca digestibility was greater in Ca-formate- than in limestone-containing diets might also be a consequence of the high Ca solubility of Ca-formate, resulting in free Ca being absorbed before it enters the small intestine (González-Vega et al., 2014). Based on the exponential model for the concentration of ATTD Ca, the increased Ca digestibility in the Ca-formate diets was independent of the presence of phytase. Recent data from growing pigs fed Ca-formate or limestone as the main Ca source in diets without or with 1,500 FTU of phytase yielded similar results for Ca and P digestibility (Klein et al., 2024). This not only corroborates the present findings but also suggests that the effect of Ca-formate solubility on mineral digestibility is not specific to young pigs. Data also suggest that the dietary Ca level in the NC diets was relatively low, as the concentration of ATTD Ca in the phytase-supplemented diets did not reach that in the PC diets. The observation that the AJD of P did not follow the same pattern as the AID and ATTD of P may indicate that most of the Ca-P and Ca-phytate complex formation occurs in the distal small intestine, where pH is the highest. This hypothesis, however, needs to be validated. Overall, results from the modeling of the dietary ATTD Ca and P concentration demonstrated that increasing concentrations of phytase in piglet diets improves the digestibility of Ca and P, regardless of the primary Ca source, but the efficiency of phytase to degrade PP and release P is influenced by the Ca solubility of the Ca source. This is in agreement with data from broiler chickens (Walk et al., 2012) and suggests that the digestibility of Ca in pig diets should be considered by formulating diets based on digestible Ca to provide the right amount of Ca to pigs and prevent a reduction in P digestibility. When the digestibility of Ca is not considered, a greater inclusion of phytase is required (1,944 instead of 1,232 FYT) in diets with Ca-formate instead of limestone as the primary Ca source.

The observation that, on average, the urinary P output of piglets was twice as high in pigs fed limestone diets as pigs fed Ca-formate diets (0.77 vs. 0.39 g, respectively) may indicate that part of the digested P in piglets fed diets with limestone was above the animal requirement or in imbalance with the digested Ca. It is well known that the presence of both Ca and P in adequate quantities and in proper ratios are needed for bone mineralization to take place (Veum, 2010). Thus, it is possible that the excess absorbed P was not retained and therefore, excreted via urine. Klein et al. (2024) reported no significant effect of Ca source (limestone or Ca-formate) on the amount of P in urine in growing pigs, although, numerically, pigs fed the limestone diets excreted twice as much P through urine as pigs fed the Ca-formate diets. This further supports previous data indicating that the main site of Ca regulation in pigs is the kidneys (González-Vega et al., 2016). Nevertheless, the influence of Ca source on the amount of P retained by piglets was limited and resulted in a slightly greater (+1.2%) P retention for piglets fed limestone than their counterparts. This indicates that replacing limestone with Ca-formate in piglet diets negatively affects P digestibility but minimally influences P retention. In the present trial, Ca was not analyzed in urine, as the main objective was to evaluate the effect of the primary dietary Ca source on P metabolism and the efficiency of phytase to release P. However, it is acknowledged that urinary Ca would have provided a complete understanding of Ca metabolism as affected by Ca source, and its inclusion is recommended in future studies with similar objectives.

The lack of a response of high doses of phytase on bone ash was not expected but might indicate that a longer adaptation period to the diets is needed to observe the results of P and Ca digestibility reflected on bone mineralization. Data from Lagos et al. (2021b) in weaned piglets support this hypothesis, because no effect of phytase supplementation to Ca- and P- adequate or deficient diets was observed on bone ash after a 15-d adaptation period. It is likely that piglets need at least 4 wks of being fed the experimental diets to exhibit phytase effects on bone mineralization, but this hypothesis needs validation. The observation that the Ca source and dietary treatment had a limited effect on the concentration of serum Ca was anticipated as Ca homeostasis is tightly regulated by hormones (Crenshaw, 2001). The values obtained in the present trial are within the physiological range of Ca in serum of pigs (80 to 120 mg/liter; Amundson et al., 2017). On the contrary, P is loosely regulated, as indicated by the positive effect of phytase supplementation observed on serum P of piglets. These results also indicated that phytase supplementation increases absorption and serum concentration of P regardless of the Ca source. Results for plasma inositol indicated that as phytase increases in the diets, the amount of phytate being fully degraded also increases. Data also suggested that the concentration of inositol in plasma becomes higher as piglets fed diets with phytase grow. Inositol is the final breakdown product of phytate, produced through the action of phytase in the digestive system of pigs. It is present in sow milk (Tan et al., 2018), can be synthesized *de novo* from glucose, and plays an essential role in various physiological processes by participating in cell signaling pathways (Huber, 2016). When plasma inositol results were analyzed throughout time, it was observed that at weaning, the concentration of inositol in plasma was maintained within a relatively narrow range, but after a week, only piglets fed diets with 1,500 or 3,000 FYT were able to maintain the initial plasma inositol level. After 18 days, the plasma inositol level in piglets fed diets without phytase had dropped to half of the level at weaning, while piglets supplemented with phytase maintained plasma inositol concentrations within or above the initial range observed. These results are well in line with those reported by Lagos et al. (2021a), who demonstrated that the concentration of plasma inositol dramatically drops in pigs fed diets without phytase during the first 4 wks after weaning, and rises to the value at weaning around week 6 post-weaning. After weaning, glucose is an important energy source to support growth and health, which likely leads to a reduced *de novo* synthesis of inositol in pigs. This, along with the removal of inositol from milk, is likely the reason for the clear reduction in plasma inositol concentrations post-weaning, as well as the increased dependency on phytase to break down phytate and release inositol. This hypothesis, however, requires further validation. Increased plasma inositol has been associated with improved feed efficiency in weaned pigs (Moran et al., 2019; Lagos et al., 2021a).

## Conclusions

Replacing limestone with Ca-formate as the primary Ca source in weaner diets reduced the dietary ABC-4, but did not affect stomach pH. Nevertheless, using Ca-formate negatively impacted the digestibility of P while improving the digestibility of Ca in diets, indicating that the high solubility of Ca in Ca-formate was the driving factor rather than its low ABC-4. The efficiency of phytase was also influenced by the Ca source as indicated by the exponential model on the concentration of ATTD P demonstrating that to reach the same dietary level of digestible P, a greater dose of phytase is needed in the Ca-formate diet than in the limestone diets. Phosphorus retention and concentration of Ca, P, and inositol in blood were less sensitive to Ca source and were mainly influenced by the dietary treatment. The practical implication of these results is that the digestibility of Ca in feed ingredients should be considered in diets to avoid Ca oversupply and its negative impact on P digestibility. Otherwise, when diets are formulated based on total Ca, a greater phytase inclusion is needed in the Ca-formate diets than in the limestone diets.

## Supplementary Material

skaf396_Supplementary_Data

## Data Availability

The data presented in this study are available on request from the corresponding author.

## Abbreviations:

ABC: acid binding capacity
AID: apparent ileal digestibility/apparent ileal digestible
AIJ: apparent jejunal digestibility
ATTD: apparent total tract digestibility/apparent total tract digestible
BW: body weight
Ca-formate: calcium formate
FYT: phytase units per kilogram feed
MCP: monocalcium phosphate
PP: phytate P
STTD: standardized total tract digestible
