# Supplementary material for: Effects of primary calcium source on phytase efficiency in weaned piglets
Source: J Anim Sci. 2025 Nov 12;103:skaf396. doi: 10.1093/jas/skaf396 (PMC12657462; doi:10.1093/jas/skaf396)
Supplement: skaf396_Supplementary_Data [file skaf396_supplementary_data.docx]

Figure 1. Fitted exponential points of the average apparent total tract digestibility (ATTD) of Ca in diets containing limestone [Y= 79.98 + (-17.62 × 0.998 ^X)^) ; R^2^= 71.7; *P* < 0.01] or Ca-formate [Y= 82.82 + (-16.97 × 0.998 ^X)^); R^2^= 66.3; *P* < 0.01] as a function of phytase inclusion level. The maximum response for the limestone diets was 79.98% (SE= 1.76), the coefficient that escalates the exponential term was -17.62 (SE= 2.52), and the growth rate was 0.998 (SE= 0.00092). The maximum response for Ca-formate diets was 82.82% (SE= 1.83), the coefficient that escalates the exponential term was -16.97 (SE= 2.65), and the growth rate was 0.998 (SE= 0.00087).

Figure 2. Fitted exponential points of the average apparent total tract digestibility (ATTD) of P in diets containing limestone [Y= 82.27 + (-32.27 × 0.998 ^X)^); R^2^= 95.4; *P* < 0.01] or Ca-formate [Y= 78.02 + (-28.50 × 0.998 ^X)^) ; R^2^= 92.9; *P* < 0.01] as a function of phytase inclusion level. The maximum response for the limestone diets was 82.27% (SE= 1.30), the coefficient that escalates the exponential term was -32.27 (SE= 1.68), and the growth rate was 0.998 (SE= 0.00021). The maximum response for Ca-formate diets was 78.02% (SE= 1.25), the coefficient that escalates the exponential term was -28.50 (SE= 1.76), and the growth rate was 0.998 (SE= 0.00031).
